# Potential application of hydrogen in traumatic and surgical brain injury, stroke and neonatal hypoxia-ischemia

**DOI:** 10.1186/2045-9912-2-11

**Published:** 2012-04-19

**Authors:** Jan M Eckermann, Paul R Krafft, Lorelei Shoemaker, Robert E Lieberson, Steven D Chang, Austin Colohan

**Affiliations:** 1 Department of Neurosurgery, Stanford University School of Medicine, Stanford, California, USA; 2 Department of Radiation Oncology, Stanford University School of Medicine, Stanford, California, USA; 3 Department of Physiology, Loma Linda University School of Medicine, Loma Linda, California, USA; 4 Department of Neurosurgery, Loma Linda University School of Medicine, Loma Linda, California, USA; 5 Department of Neurosurgery, Stanford Hospital and Clinics, 300 Pasteur Drive, Room R281, Stanford, CA 94304, USA

**Keywords:** Hydrogen, Neuroprotection, Oxidative stress, Reactive oxygen species

## Abstract

This article summarized findings of current preclinical studies that implemented hydrogen administration, either in the gas or liquid form, as treatment application for neurological disorders including traumatic brain injury (TBI), surgically induced brain injury (SBI), stroke, and neonatal hypoxic-ischemic brain insult (HI). Most reviewed studies demonstrated neuroprotective effects of hydrogen administration. Even though anti-oxidative potentials have been reported in several studies, further neuroprotective mechanisms of hydrogen therapy remain to be elucidated. Hydrogen may serve as an adjunct treatment for neurological disorders.

## Introduction

Traumatic brain injury (TBI) and cerebral vascular events can be devastating; yet there are few treatments proven to ameliorate the brain damage and overall outcome in patients. An ideal neuroprotectant would be non-toxic, easily administered, permeable at the blood-brain barrier (BBB), and offer protection at all stages of injury, including prophylaxis. Hydrogen sulfide (H_2_S) has shown some of these properties [1,2]. Its mechanisms may be related to the attenuation of reactive oxygen species (ROS) [3], or to its role as a neuromodulator [4]; however, the use of H_2_S is controversial because of its toxicity [5] and gasotransmitter functions [6].

Hydrogen is the most abundant element in the universe [7]. Room air concentrations of hydrogen gas higher than 4% (normal air content = 0.000055%) are explosive, and could cause asphyxiation [8]. Therapeutically relevant dosages of hydrogen, as used in the following animal studies (range 2%-2.9%), appear to be well tolerated. Furthermore, 3% hydrogen gas has been safely and regularly used for human deep-sea divers without any adverse events [9].

Currently, to our knowledge, there are no FDA approved therapeutic regimens involving hydrogen gas or dissolved hydrogen. We sought to analyze recent available data regarding the use of hydrogen as a neuroprotectant.

### Hydrogen therapy for traumatic brain injury and cerebral vascular events

#### Traumatic brain injury

Trauma is the leading cause of death in Americans younger than 45 years of age, and traumatic brain injury (TBI) accounts for over 50% of this mortality [10]. Out of the 1.5 million Americans who sustain a TBI each year, 230,000 are hospitalized, 80,000 to 90,000 remain with long-term disabilities, 50,000 die, and the estimated annual cost exceeds $60 billion [11-13]. To investigate whether hydrogen (H_2_) exerts abilities to ameliorate the outcome after TBI, Ji et al. administered 2% H_2 _to rats, which were also subjected to experimental TBI [14]. TBI-challenged rats, that did not inhale H_2_, presented with significantly greater brain edema, blood-brain barrier (BBB) permeability, lesion size, and neurological impairments, than those in the treatment group. Decreased levels of oxidative products such as malondialdehyde (MDA) and 8-iso-prostaglandin F2α (8-iso-PF2α), were decreased in brain tissues of treated animals, suggesting that H_2 _induces neuroprotection by reducing oxidative stress to the BBB.

#### Surgical brain injury

Each year, approximately 800,000 patients undergo neurosurgical procedures in the United States [15]. This figure encompasses emergent as well as planned procedures. Neurosurgical operations have the potential to cause unavoidable damage to healthy brain tissue through application of pressure, tissue stretching, hemorrhage, and the use of electrocautery [16]. This form of brain injury occurs regularly, despite of intraoperative adjunct treatments such as administration of steroids and mannitol in both emergent or scheduled procedures [17]. Even standard microsurgical techniques have the ability to cause damage to the surrounding brain tissue, thus leading to early complications such as edema formation and local ischemia.

To determine the effects of hydrogen treatment following surgically-induced brain injury (SBI), Eckermann et al. utilized a novel rat model that involved a partial right frontal lobectomy [18]. The following groups were compared: rats subjected to sham operation (craniotomy), SBI animals that received 2.9% hydrogen, which was administered concurrently with surgery for a period of 0.5 hours, and a control group (SBI + room air). Brain water content and neurological deficits were measured at 24 hours after SBI. The results demonstrated that hydrogen treatment significantly decreased the formation of cerebral edema, which resulted in improved neurobehavioral function; however, the treatment failed to reduce oxidative stress and cerebral inflammation (evaluated via Lipid Peroxidase and Myeloperoxidase assays, respectively) in rats subjected to SBI.

#### Stroke

Stroke is a leading cause of death and long-term disability, particularly in the elderly population [19]. It is associated with a 30-day mortality rate of approximately 20% [19]. The prevalence of stroke is expected to increase significantly as the global population of men and women - older than 65 years of age - increases continuously by an estimated 9 million people per year [20]. Consequently, it is most essential to investigate novel and potentially effective therapeutics to improve the neurofunctional outcome in patients. The effects of hydrogen treatment for ischemic stroke have been evaluated in an experimental rodent model of transient middle cerebral artery occlusion (tMCAO) [21]. This model exerts its damaging effect through focal ischemia and reperfusion, which generates acute oxidative stress to the affected brain regions. Under general anesthesia the rat's left middle cerebral artery (MCA) was occluded using a nylon monofilament with a distal silicon rubber tip. The treatment group inhaled 2% hydrogen gas during the entire procedure. Treated and control animals underwent neurological testing, and were sacrificed at 12 hours, 24 hours, 3 days, and 7 days post-surgery. Brains were then sectioned and stained with 2,3,5-triphenyltetrazolium chloride (TTC) to label the infarcted brain area, followed by volumetric computation of the infarct volume. Observed protective effects of hydrogen therapy included: decreased infarct volume, maintenance of body weight after surgery, and improved neurological function when compared to control animals. Liu et al. [22] utilized the tMCAO model to evaluate neuroprotective effects of intraperitoneally administered hydrogen saline (1 ml/100 g body weight). The results showed that hydrogen saline significantly reduced infarct volume, brain edema, and neurological function when administered within a 6 hour time window after ischemia induction. Hydrogen saline reduced ROS, inflammation markers, as well as caspase 3 activity in the ischemic brain [22]. Matchett et al. administered hydrogen gas (2.9%) to adult rats that were subjected to tMCAO [23]. Hydrogen administration showed a tendency to reduce the infarction volume in the treatment group, when compared to control animals; however, neurological deficits were similar in both groups. Furthermore, Chen et al. [24] reported that hydrogen gas effectively reduced acute hyperglycemia-enhanced hemorrhagic transformation after focal ischemia in the rat. Potential mechanisms on how hydrogen gas ameliorates hemorrhagic transformation remain to be investigated.

Oshawa et al. demonstrated that hydrogen selectively reduced ROS in neuronal tissue cultures [21]. The effects were demonstrated via electron spin resonance signals in cells subjected to oxygen glucose deprivation followed by oxygen glucose reperfusion, which models cerebral ischemia-reperfusion in vitro [21]. After reperfusion, fluoroscopy demonstrated an immediate decrease in hydroxyl radicals (OH^-^), and an increase in neuronal survival at 24 hours, suggesting that hydrogen effectively protects neurons from oxidative stress-mediated cell death.

Hemorrhagic stroke is often more severe than ischemic stroke, and includes intracerebral hemorrhages (ICH) and subarachnoid hemorrhages (SAH). The mortality rates for ICH and SAH have been reported as 40% to 50%, and survivors are commonly affected by chronic morbidity [25]. Furthermore, the aggregate lifetime cost of hemorrhagic stroke cases has been estimated as high as $5.6 to $6.0 billion per year [26]. The effects of hydrogen administration in hemorrhagic stroke models have been previously reported. In a mouse model that implemented collagenase-induced ICH, hydrogen therapy was found to significantly reduce cerebral edema and neurological deficits at 24 hours after surgery; however, hydrogen post-treatment showed only a tendency to improve these outcomes at 72 hours after surgery [27]. The authors conclude that hydrogen inhalation exerts an acute neuroprotective effect in mice subjected to experimental ICH. Similarly to these results, hydrogen treatment (2.9% for 2 hours) starting 1 hour after induction of experimental SAH (endovascular perforation method), ameliorated brain edema, reduced apoptosis, and improved neurological deficits at 24 hours, but not at 72 hours after surgery [28]. The protective effects of hydrogen therapy were associated with the reduction of oxidative injury of lipids, proteins, and DNA.

#### Neonatal hypoxia-ischemia

Advances in perinatal care and neonatology led to increased survival of premature infants [29], yet the incidence of brain injury caused by neonatal hypoxia-ischemia (HI) remains high [30]. Effective therapeutic modalities are limited for the treatment of HI. This form of CNS injury occurs at a very early stage in life. It is accompanied by a heavy toll on families and significant long-term healthcare demands. Since neonates have an immature free radical buffering system, they are believed to be more vulnerable to ROS damage.

Hydrogen treatment was associated with improved outcomes in a model of neonatal hypoxia/ischemia [31,32]. Cai et al. [32] subjected seven-day old rat pups to left common carotid artery ligation followed by 90 minutes of hypoxia. Immediately after the HI injury the pups were placed in chambers containing 2% hydrogen for 30, 60, and 120 minutes. Twenty-four hours following hydrogen therapy, the animals were sacrificed and the brain damage was assessed with TTC and Nissl staining, TUNEL, and evaluation of caspase activity. Hydrogen therapy significantly reduced the number of TUNEL positive cells, and attenuated caspase activity, suggesting that hydrogen gas exerts neuroprotective effects by inhibiting cellular apoptosis after HI. However, in another study of HI, hydrogen treatment failed to show neuroprotective effects, possibly as a result of the severity of the injury [23]. The authors concluded that hydrogen gas may have a neuroprotective effect against mild to moderate HI, but loses that effect in cases of severe HI injury.

## Discussion

Despite significant efforts directed towards the development of therapies that effectively reduce brain damage, particularly in TBI, only moderate success has been reported. So far, most advances involved preventative behavior modifications, such as wearing seat belts and helmets when using motor vehicles and motorcycles [12,13]. Further efforts need to be directed towards the search of neuroprotective agents that effectively improve the outcome of patients, even when applied post-injury. Many approaches have been suggested as post-treatments for CNS injuries such as hypothermia, vitamin E, hyperbaric oxygen, and numerous medications for ischemic and hemorrhagic strokes [33,34]. Post-injury protection reducing subsequent progression of the brain damage is critical; however, current success has been limited at best. Continued efforts are warranted to identify suitable neuroprotective agents that are inexpensive, easy to administer, and that present high therapeutic indexes. Increasing evidence suggests that hydrogen may be such an agent (Table 1). Several studies claim that hydrogen evokes cellular protection through attenuation of ROS production following diverse brain injuries. It has been demonstrated that hydrogen exerts a therapeutic antioxidant effect by selectively reducing hydroxyl radicals (OH^-^), which are believed to be potent cytotoxic ROS, indiscriminately damaging proteins, lipids, and nucleic acids [21]. Furthermore, no known cellular detoxification system exists for OH^-^. Hydrogen also effectively protects cellular damage to non-neuronal tissues including renal, lung, and myocardial injury [21].

**Table 1:** Animal studies examining neuroprotection by hydrogen

Author	Year	Model	Species	Effects of H_2_	Proposed Mechanism
Ji et al. [14]	2010	TBI	Rat	Brain edema↓, neurological deficits↓	H_2 _increased endogenous antioxidant enzymatic activities
Eckermann et al. [18]	2011	SBI	Rat	Brain edema↓, neurological deficits↓	None proposed
Ohsawa et al [21]	2007	tMCAO	Rat	Brain infarction↓, neurological deficits↓	H_2 _selectively inhibited OH^-^
Matchett et al. [23]	2009	tMCAO	Rat	Tendentially reduced brain infarction	None proposed
Chen et al. [24]	2010	tMCAO	Hyperglycemic rat	Hemorrhagic transformation↓	H_2 _reduced hyperglycemia
Liu et al. [22]	2011	tMCAO	Rat	Brain edema↓, infarction↓, neurological deficits↓	ROS↓, inflammation↓, apoptosis↓
Manaenko et al. [27]	2011	ICH	Mouse	Acute brain edema↓, neurological deficits↓	None proposed
Zhan et al. [28]	2012	SAH	Rat	Acute brain edema↓, neurological deficits↓	BBB permeability↓, ROS↓, apoptosis↓
Matchet et al. [23]	2009	HI	Mouse	No beneficial effects	None proposed
Cai et al. [31]	2008	HI	Rat	Brain infarction↓, apoptosis↓	H_2 _selectively inhibited caspase activity

Utilized models include traumatic brain injury (TBI), surgically induced brain injury (SBI), transient middle cerebral artery occlusion (tMCAO), intracerebral hemorrhage (ICH), subarachnoid hemorrhage (SAH) and neonatal hypoxia-ischemia (HI)

A recent clinical study by Ono et al. [35] showed that acute treatment with OH^- ^scavengers (Edaravone and hydrogen) improved MRI indices (accelerated normalization) in patients with brainstem infarction. In fact, intravenous injection of Edaravone combined with hydrogen saline showed significantly better results than administration of Edaravone alone. The effects of hydrogen have also been examined in a randomized, double-blinded, placebo-controlled crossover study implemented in patients with diabetes mellitus type 2 [36]. Patients that received 900 ml/day of hydrogen-rich water for 8 weeks showed significant improvements of LDL cholesterol and urinary 8-isoprostanes. Further protective effects of hydrogen therapy were found in patients with mitochondrial myopathies, polymyositis and dermatomyositis [37].

Importantly, it needs to be determined if hydrogen treatment alters normal metabolism, since several agents classified as ROS, such as nitric oxide (NO), have important cell signaling functions and are regulators of physiologic responses [21]. It is crucial that the administered antioxidant does not disturb these normal cell-signaling pathways. Hydrogen may also function by preventing break-down of the BBB. The most prominent and consistent effect is the reduction of cerebral edema and the improvement of neurological function post-injury. Hydrogen has several advantages as a potential antioxidant: it effectively reduces cytotoxic OH^- ^in vivo, and demonstrates excellent penetration characteristics. Its ability to enter biomembranes and diffuse into mitochondria and cell nuclei render it highly effective as a powerful antioxidant [21].

Taken together, hydrogen appears to be a safe therapeutic with the ability to attenuate ROS production after diverse CNS injuries. Furthermore, hydrogen effectively reduced neuronal apoptosis and hemorrhagic transformation after ischemic brain injury. Its ease of use and availability will facilitate clinical transition in the near future; however, further mechanisms of neuroprotection need to be primarily investigated.

## Abbreviations

BBB: Blood brain barrier; H_2_: Hydrogen; H_2_S: Hydrogen sulfide; HI: Neonatal hypoxia-ischemia; ICH: Intracerebral hemorrhage; ROS: Reactive oxygen species; MCA: Middle cerebral artery; SAH: Subarachnoid hemorrhage; TBI: Traumatic brain injury; tMCAO: Transient middle cerebral artery occlusion; TTC: 2:3:5-triphenyltetrazolium chloride.

## Competing interests

The authors declare that they have no competing interests.

## Authors' contributions

JE initiated this study. He conducted the literature review together with PK, LS, RL and SD. JE and PK provided review of the use of hydrogen in traumatic and surgically induced brain injury. LS, RL focused on ischemic and hemorrhagic stroke. SD provided review of the use of hydrogen in neonatal hypoxia-ischemia. All authors discussed their individual findings with all co-authors and collectively developed the discussion section of this paper. AC supervised all meetings for discussion and made substantial contributions to the overall content. AC also edited this paper. All authors have read and approved the final manuscript.
